# Mitochondrial efficiency and insulin resistance

**DOI:** 10.3389/fphys.2014.00512

**Published:** 2015-01-05

**Authors:** Raffaella Crescenzo, Francesca Bianco, Arianna Mazzoli, Antonia Giacco, Giovanna Liverini, Susanna Iossa

**Affiliations:** Department of Biology, University of Naples “Federico II”Napoli, Italy

**Keywords:** mitochondria, type 2 diabetes, insulin, skeletal muscle, proton leak

## Abstract

Insulin resistance, “a relative impairment in the ability of insulin to exert its effects on glucose, protein and lipid metabolism in target tissues,” has many detrimental effects on metabolism and is strongly correlated to deposition of lipids in non-adipose tissues. Mitochondria are the main cellular sites devoted to ATP production and fatty acid oxidation. Therefore, a role for mitochondrial dysfunction in the onset of skeletal muscle insulin resistance has been proposed and many studies have dealt with possible alteration in mitochondrial function in obesity and diabetes, both in humans and animal models. Data reporting evidence of mitochondrial dysfunction in type two diabetes mellitus are numerous, even though the issue that this reduced mitochondrial function is causal in the development of the disease is not yet solved, also because a variety of parameters have been used in the studies carried out on this subject. By assessing the alterations in mitochondrial efficiency as well as the impact of this parameter on metabolic homeostasis of skeletal muscle cells, we have obtained results that allow us to suggest that an increase in mitochondrial efficiency precedes and therefore can contribute to the development of high-fat-induced insulin resistance in skeletal muscle.

Obesity and the related metabolic disorders, such as insulin resistance and type 2 diabetes, are growing dramatically all over the world, so that experts are predicting an “obesity pandemic.” In particular, it has been estimated that in year 2030 about 400 million people will exhibit type 2 diabetes (Wild et al., 2004). This alarming scenario arises from the prevalence of factors like consumption of high-fat diets and low physical activity.

Because the terms “insulin resistance,” “type 2 diabetes” and “mitochondrial efficiency” are central to this discussion, their definition is fundamental. Insulin resistance is defined as “a relative impairment in the ability of insulin to exert its effects on glucose, protein and lipid metabolism in target tissues,” so that at physiological concentrations insulin produces a lower biologic response (Kahn, 1978). Therefore, insulin resistance has many detrimental effects on metabolism that are the basis for a number of chronic diseases, including type 2 diabetes, a metabolic disorder of multiple etiology characterized by chronic hyperglycemia with disturbances of carbohydrate, fat, and protein metabolism resulting from defects in insulin secretion, insulin action, or both (Alberti and Zimmet, 1998). One fundamental process in the mitochondria is the oxidative phosphorylation, in which the electrons are removed from organic molecules and transferred to oxygen and the energy released is used in the synthesis of ATP. The amount of ATP formed per unit of consumed oxygen is determined by the efficiency of oxidative phosphorylation (Mogensen and Sahlin, 2005).

One of the most deleterious effects of obesity is deposition of lipids in non-adipose tissues, such as liver, skeletal muscle, and heart. It has been proposed that the accumulation of lipids in the muscle cell should interfere with insulin signaling, thereby causing insulin resistance. In agreement with this hypothesis, a strong association between fat accumulation in skeletal muscle (and liver) and insulin resistance has been found in men (McGarry, 2002). In addition, high levels of intramyocellular lipids (IMCL) and muscular insulin resistance have been found in type 2 diabetic patients (Goodpaster et al., 2001) and in high-risk non-diabetic subjects with a family history of diabetes (Jacob et al., 1999; Perseghin et al., 1999). However, high IMCL levels do not necessarily lead to insulin resistance, since they are also present in skeletal muscle from endurance-trained athletes, who are highly insulin-sensitive (Goodpaster et al., 2001; Schrauwen-Hinderling et al., 2006). The emerging idea is that increased intramuscular fat turns to be deleterious when an increase in the supply of lipids to skeletal muscle is not balanced by an increase in the oxidative pathways, so that toxic intermediates, such as ceramides and diacylglycerol, accumulate in the cell and interfere with the insulin signaling system (Kelley and Mandarino, 2000; Shulman, 2000). Therefore, in the above picture, a prominent role is played by the level of cellular oxidative capacity of fatty acids.

Mitochondria are the main cellular sites devoted to fatty acid oxidation. Therefore, a role for mitochondrial dysfunction in the onset of skeletal muscle insulin resistance has been proposed and several studies have dealt with possible alteration in mitochondrial function in obesity and diabetes, both in humans and animal models.

Studies in humans have shown that type 2 diabetes patients exhibited alteration in mitochondrial morphology, as well as a decrease in the activity of the respiratory chain (Kelley et al., 2002; Ritov et al., 2010). Other studies showed a coordinated reduction in the expression of genes encoding key enzymes in oxidative mitochondrial metabolism in diabetic patients and in high-risk non-diabetic subjects with a family history of diabetes (Mootha et al., 2003; Patti et al., 2003). Petersen et al. (2003) reported a 40% decrease in oxidative metabolism in elderly subjects, that were also characterized by elevated levels of muscular fat and by muscular insulin resistance, thus suggesting that an age-associated decline in mitochondrial function might contribute to the development of insulin resistance. They also found that IMCL and ATP synthase were, respectively, 80% higher and 30% lower in insulin resistant subjects (Petersen et al., 2004). Szendroedi et al. (2007) found that *in vivo* ATP synthesis rate was decreased by 27% in diabetic patients, while in other studies *in vivo* mitochondrial function was compromised by ~ 45% in type 2 diabetic patients, although IMCL content was similar between the groups, suggesting that impaired mitochondrial function may be a more important determinant of diabetes than IMCL levels (Schrauwen-Hinderling et al., 2007; Phielix et al., 2008). Taken together, these studies are consistent in showing that *in vivo* mitochondrial function is reduced in insulin resistant subjects and/or type 2 diabetic patients. This decrease could lead to accumulation of fat in muscle, but also provide lesser amount of ATP for membrane transports and signal transduction pathways, thereby contributing to the development of insulin resistance.

However, other observations argue against the hypothesis that mitochondrial dysfunction underlies the development of type 2 diabetes mellitus or muscular fat accumulation (Hancock et al., 2008; Han et al., 2011). In fact, several studies present findings in support of the concept that muscular fat accumulation may precede the development of mitochondrial dysfunction and/or that insulin resistance arises when mitochondrial function is unaffected or even improved (Turner et al., 2007; Hoeks et al., 2008; Ara et al., 2011). For example, an improved or unchanged mitochondrial oxidative capacity has been found after consumption of a high-fat diet in mice or rats exhibiting insulin resistance (Turner et al., 2007; Hoeks et al., 2008). These data suggest that high-fat diets, although leading to insulin resistance in rodents, are not accompanied by mitochondrial dysfunction, but rather they lead to improved mitochondrial oxidative capacity. Other researchers looked at the time course of changes in mitochondrial function in skeletal muscle in response to high-fat feeding. Chanseaume et al. (2007) showed a transiently enhanced activity of the oxidative phosphorylation after 14 days, but a significant decrease at day 40. Laurent et al. (2007) showed in rats that ATP synthesis rates decreased by 50% within 24 h, returned to normal values after 2–3 weeks on the high-fat diet, and again decreased by 30–50% after 1 month. Finally, Bonnard et al. (2008) showed that 1 month of high-fat, high-sucrose diet feeding induced glucose intolerance in mice, without mitochondrial dysfunction, that was evident after 16 weeks. Taken together, these studies are consistent with the hypothesis that mitochondrial dysfunction may be a consequence rather than cause of muscular fat accumulation, but this does not exclude the possibility that mitochondrial dysfunction could in turn induce insulin resistance.

Physical activity is a major regulator of mitochondrial function in muscle, and exercise potently activates mitochondrial biogenesis, while chronic inactivity is associated with reduced mitochondrial number (Hoppeler and Fluck, 2003; Little et al., 2011). Obesity and other metabolic disorders are linked with reduced activity levels and increased sedentary behavior (Hamilton et al., 2007; Levine et al., 2008). Thus, it is possible that some mitochondrial defects reported in overweight or obese insulin-resistant subjects can be explained, in part, by low levels of physical activity. In this respect, animal models are very useful tools, since rats kept in laboratory display a sedentary behavior, due to standard housing conditions (Spangenberg et al., 2005), and therefore it is possible to perform studies aiming at the elucidation of the link between insulin resistance and mitochondrial functioning, without the confounding effect of changes in physical activity. Another possible reason of the apparent discrepancy among the various results published on the above issue is the choice of the parameter to be studied in evaluating mitochondrial function. In fact, if the hypothesis is that reduced mitochondrial oxidation of fatty acids causes ectopic fat deposition, that in turn elicits insulin resistance, all the factors contributing to mitochondrial lipid burning must be taken into account. The mitochondrial oxidation of metabolic fuels depends not only on organelle number and organelle activity, but also on energetic efficiency of the mitochondrial machinery in synthesizing ATP from the oxidation of fuels (Figure 1). Changes in each of these three factors could theoretically affect lipid oxidation and should be monitored to confirm or reject the hypothesis (Figure 1).

**Figure 1:**
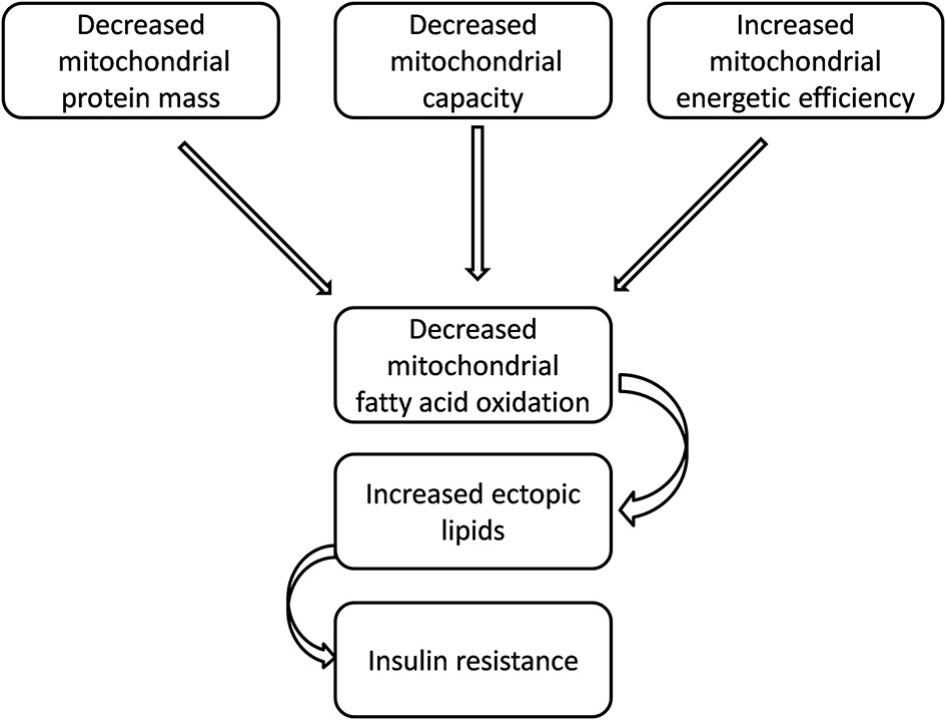
**A possible link between insulin resistance and impaired mitochondrial performance**.

Many reported studies on the issue of mitochondrial dysfunction in insulin resistant states have focused the attention on mitochondrial impairment in terms of reduced mass and/or oxidative activity. However, it is well-known that the amount of fuels oxidized by the cell is dictated mainly by ATP turnover rather than by mitochondrial oxidative activity (Boveris et al., 2000) and therefore, in resting skeletal muscle, changes in organelle number and/or activity could be without consequence for cellular bioenergetics, while modifications in mitochondrial energetic efficiency certainly alter the amount of oxidized fuels, even if ATP turnover does not vary. In fact, the efficiency with which dietary calories are converted to ATP is determined by the coupling efficiency of oxidative phosphorylation. If the respiratory chain is highly efficient at pumping protons out of the mitochondrial inner membrane, and the ATP synthesis is highly efficient at converting the proton flow through its proton channel into ATP (from ADP), then the mitochondria will generate maximum ATP and minimum heat per calorie. These mitochondria are said to be “tightly coupled.” In contrast, if the efficiency of proton pumping is reduced and/or more protons are required to make each ATP molecule, then each calorie will yield less ATP but more heat. Such mitochondria are said to be “loosely coupled.” Therefore, the coupling efficiency determines the balance of calories used to perform work (ATP) or for heat generation. It remains to be established whether, under high-fat conditions, cellular ATP demand is altered, a parameter that can only be assessed in the living animal (Amara et al., 2008).

To our knowledge, data on the energetic efficiency in skeletal muscle mitochondria in conditions of obesity-induced insulin resistance are scarce. By using a rat model of high-fat diet-induced obesity, we have evidenced that after 1 and 2 weeks of high-fat feeding (Crescenzo et al., 2014a,b), skeletal muscle mitochondrial efficiency is increased, thus giving rise to a reduced burning of energy substrates. This modification of mitochondrial efficiency takes place at a time point when insulin sensitivity is still maintained (Crescenzo et al., 2014a,b). Therefore, these results could be consistent with a role for mitochondrial impairment in the onset of insulin resistance. In fact, if mitochondria are more coupled, less substrates need to be burned to obtain the same amount of ATP. At the same time, high-fat feeding is associated with increased lipid supply to skeletal muscle (Crescenzo et al., 2014b), so that a condition of imbalance could occur, since lipid supply exceeds lipid burning and gives rise to ectopic lipid deposition. In agreement with this suggestion, we have also found increased levels of skeletal muscle triglycerides (Crescenzo et al., 2014b). Interestingly, when high-fat diet intake was carried out for 7 weeks, insulin resistance developed but the alteration in mitochondrial efficiency disappeared (Lionetti et al., 2007). One possible explanation could be related to changes in lipid composition of the mitochondrial membranes induced by the high-fat intake, one of the factors contributing to mitochondrial proton leak and hence to mitochondrial efficiency (Jastroch et al., 2010).

A similar increase in mitochondrial efficiency is also evident after 2 weeks of feeding a high-fat-high fructose diet (Crescenzo et al., 2014b) but in the presence of insulin resistance. Since mitochondrial energetic efficiency is higher both in rats with normal insulin sensitivity (high-fat-fed rats) and in those with decreased insulin sensitivity (high-fat-high fructose-fed rats) we can hypothesize that this mitochondrial modification is not caused by, but could contribute to, the onset of insulin resistance. In agreement with this suggestion, the content of skeletal muscle ceramides (known to be mediators of altered insulin signaling, Coen and Goodpaster, 2012) is higher in rats fed high-fat diet but even higher in rats fed high-fat-high fructose diet and therefore it is possible that in the latter group of rats its concentrations have reached a threshold level to be able to partly block insulin transduction pathway. In addition, in rats fed a low-fat, fructose-rich diet we have found a reduced insulin signaling system in skeletal muscle concomitant to an increase in mitochondrial efficiency and cellular levels of ceramides after 8 weeks of dietary treatment (Crescenzo et al., 2013).

In support of the link between mitochondrial efficiency and insulin resistance in skeletal muscle during high-fat feeding are the results showing that the naturally occurring iodothyronine, 3,5-diiodo-L-thyronine (T2) increases mitochondrial proton leak (Lombardi et al., 2007, 2009), thus decreasing mitochondrial efficiency, and when given to high-fat-fed rats is able to reverse high-fat-induced insulin resistance (de Lange et al., 2011; Moreno et al., 2011).

Another condition of obesity and related insulin resistance is the progression of aging. In agreement with results obtained on diet-induced obesity, when studying age-induced obesity in rats, we have found an increase in skeletal muscle mitochondrial efficiency, in parallel with the development of insulin resistance, in the transition from young (60 days) to middle age (180 days) (Iossa et al., 2004), and a further increase from middle age to old age (2 years) (Crescenzo et al., 2014c).

## Concluding remarks

In summary, results reporting evidence of mitochondrial dysfunction in type 2 diabetes mellitus are numerous, even though the issue that this reduced mitochondrial function is causal in the development of the disease is not yet solved, also because a variety of parameters have been used in the studies carried out on this subject. By assessing the alterations in mitochondrial efficiency as well as the impact of this parameter on metabolic homeostasis of skeletal muscle cells we have obtained results that allow us to suggest that an increase in mitochondrial efficiency precedes and therefore can contribute to the development of high-fat induced insulin resistance in skeletal muscle.

### Conflict of interest statement

The authors declare that the research was conducted in the absence of any commercial or financial relationships that could be construed as a potential conflict of interest.
